# The role of NF-κB and Elk-1 in the regulation of mouse ADAM17 expression

**DOI:** 10.1242/bio.039420

**Published:** 2019-02-15

**Authors:** Karolina Wawro, Mateusz Wawro, Magdalena Strzelecka, Maria Czarnek, Joanna Bereta

**Affiliations:** Department of Cell Biochemistry, Faculty of Biochemistry, Biophysics and Biotechnology, Jagiellonian University, Kraków 30-387, Poland

**Keywords:** ADAM17, NF-κB, Elk-1, *Adam17* promoter, Cytokines, PMA

## Abstract

ADAM17 is a cell membrane metalloproteinase responsible for the release of ectodomains of numerous proteins from the cell surface. Although ADAM17 is often overexpressed in tumours and at sites of inflammation, little is known about the regulation of its expression. Here we investigate the role of NF-κB and Elk-1 transcription factors and upstream signalling pathways, NF-κB and ERK1/2 in ADAM17 expression in mouse brain endothelial cells stimulated with pro-inflammatory factors (TNF, IL-1β, LPS) or a phorbol ester (PMA), a well-known stimulator of ADAM17 activity. Notably*,* NF-κB inhibitor, IKK VII, interfered with the IL-1β- and LPS-mediated stimulation of ADAM17 expression. Furthermore, *Adam17* promoter contains an NF-κB binding site occupied by p65 subunit of NF-κB. The transient increase in *Adam17* mRNA in response to PMA was strongly reduced by an inhibitor of ERK1/2 phosphorylation, U0126. Luciferase reporter assay with vectors encoding the ERK1/2 substrate, Elk-1, fused with constitutively activating or repressing domains, indicated Elk-1 involvement in *Adam17* expression. The site-directed mutagenesis of potential Elk-1 binding sites pointed to four functional Elk-1 binding sites in *Adam17* promoter. All in all, our results indicate that NF-κB and Elk-1 transcription factors via NF-κB and ERK1/2 signalling pathways contribute to the regulation of mouse *Adam17* expression.

## Abstract (precis)

**Summary:** We show the involvement of ERK1/2 and NF-κB pathways in the stimulation of mouse *Adam17* expression and determine functional Elk-1- and NF-κB binding sites in its promoter.

## INTRODUCTION

Inflammation is a process that allows multicellular organisms to defeat and remove invading pathogens. Although acute inflammation is a beneficial process that ensures the maintenance of tissue and organism homeostasis, chronic inflammation is a hallmark of many autoimmune diseases (Navegantes et al., 2017). A persistent inflammatory state may also lead to cancer development (Chai et al., 2015). Tumour necrosis factor (TNF) is one of the most important mediators of both the acute and chronic inflammation. This cytokine drives progression of chronic inflammatory diseases including rheumatoid arthritis, psoriasis and Crohn's disease (Aggarwal et al., 2002). Although, as its name suggests, TNF may trigger necrosis of certain tumours, it may also promote tumour progression (Sethi et al., 2008). Development of these pathologies is accompanied by elevated levels of TNF in plasma and in affected tissues (Monaco et al., 2015; Sedger and McDermott, 2014; Sethi et al., 2008).

ADAM17 (a disintegrin and metalloproteinase domain-containing protein 17), known also as TACE (TNF-alpha-converting enzyme), was identified as the main enzyme responsible for a limited proteolysis of membrane TNF precursor, which leads to the release of soluble TNF from the cell surface (Black et al., 1997; Moss et al., 1997). Nowadays more than 80 substrates of this prominent member of the ADAM family are known, among them most of the EGFR ligands and mediators of immune response including L-selectin and IL-6R (Moss and Minond, 2017; Rose-John, 2013). Since ADAM17 is the sheddase of TNF and other inflammatory proteins it is reasonable to suspect that increased levels of soluble substrates of ADAM17 would be associated with elevated ADAM17 expression and/or activity. Indeed, both the levels and activity of ADAM17 were found augmented in inflamed and/or tumour tissues (McGowan et al., 2007; Nishimi et al., 2018; Scheller et al., 2011).

The activity of ADAM17 is tightly regulated. Produced as an inactive proprotein, ADAM17, guided by its iRhom chaperones, exits ER and after proprotein convertase-mediated removal of the prodomain reaches the plasma membrane (Grötzinger et al., 2017). Here its activity may be still impeded by its interactions with α5β1 integrin (in cis) and/or tissue inhibitor of metalloproteinase-3 (TIMP3). The disruption of these interactions is necessary but not sufficient for ADAM17 activation because its activity depends on the conformation of its membrane-proximal domain (MPD) (Grötzinger et al., 2017). Diverse factors strongly enhance ADAM17 activity, among them activators of protein kinase C, agonists of purinergic receptor 2, calcium ionophores, fibroblast growth factor 7, diminished membrane cholesterol content and apoptosis (Sommer et al., 2016). All these factors share a common denominator; they influence the distribution of phosphatidylserine (PS) in the plasma membrane leading to an increased PS content in its outer leaflet. Indeed, the interaction of ADAM17 MPD with surface-exposed PS, which brings the protease domain into the right position for a substrate cleavage, was shown to be a key factor for the sheddase activation (Sommer et al., 2016). The activity of ADAM17 is also controlled by the regulation of its surface expression (Grötzinger et al., 2017).

In light of this multilayer regulation of activity a question arises whether the changes in the level of *Adam17* transcription may have any effect on ADAM17-mediated shedding. Indeed, Yoda et al. showed that systemic overexpression of ADAM17 did not result in increased plasma levels of its substrates (Yoda et al., 2013). In contrast, Fukaya et al. demonstrated that dermal fibrosis, which followed PMA-induced inflammation, was augmented in ADAM17-overexpressing mice compared with wild-type animals (Fukaya et al., 2013). Also our previous *in vitro* experiments showed increased shedding of TNFR1, a substrate of ADAM17, upon cytokine-mediated moderate stimulation of ADAM17 expression (Bzowska et al., 2004). It is possible that strong, non-physiological overexpression of ADAM17 favours its dimerization, which facilitates the interaction with TIMP3 and thus limits its activity (Grötzinger et al., 2017). Under conditions that promote ADAM17 maturation, plasma membrane localization and adaptation of an active conformation, increased transcription of *Adam17* could augment the overall activity of the sheddase. However, not much attention has been given to the regulation of ADAM17 expression via transcriptional activation.

ADAM17 is expressed in virtually all cell types, although the level of expression varies between tissues (Rose-John, 2013). However, the knowledge of the exact factors and signalling pathways that affect ADAM17 expression are still scarce. Ermert et al. showed an increase in ADAM17 protein level in rat lung endothelial cells in response to LPS (Ermert et al., 2003). TNF was shown to increase *Adam17* mRNA in endothelial cells (Bzowska et al., 2004). The effect of TNF on *ADAM17* expression was also shown in human oral squamous cell carcinoma cells (Takamune et al., 2008). The increase of *ADAM17* mRNA that was observed after 12 h of TNF-stimulation was dependent on NF-κB, because the use of the inhibitor of activation of this transcription factor – NBD (NEMO-binding domain) – caused inhibition of the observed increase (Takamune et al., 2008). The role of NF-κB in the regulation of mouse *Adam17* expression was suggested by Nikolaidis et al. (2010). They showed that alveolar macrophages isolated from Ron tyrosine kinase-deficient mice exhibit a higher expression of ADAM17 at both transcript and protein levels compared to a wild-type control. Additionally, the activation of Ron receptor in wild-type macrophages results in a pronounced decrease of the *Adam17* transcript level, indicating that the Ron receptor is a negative regulator of *Adam17* expression. On the other hand, activation of Ron leads to an inhibition of NF-κB activity, which may suggest that the negative regulation of ADAM17 protein by the Ron receptor is due to the inhibition of the NF-κB (Nikolaidis et al., 2010). Charbonneau et al. demonstrated that TNF and hypoxia regulate the expression of ADAM17 in rat synovial cells (Charbonneau et al., 2007). Hypoxia-induced ADAM17 expression was also analysed by Szalad et al., who showed that Sp1 transcription factor binds to the ADAM17 promoter in U87 human glioma cells (Szalad et al., 2009). Moreover, ADAM17 was identified as a novel target of the unfolded protein response (UPR) (Rzymski et al., 2012). The authors showed that the stimulation of ADAM17 expression by severe hypoxia in several tumour cell lines occurs as a consequence of the activation of the PERK/eIF2α/ATF4 pathway and activating transcription factor 6 (ATF6) (Rzymski et al., 2012). Also, high glucose concentration was shown to upregulate ADAM17 in mesangial cells via HIF-1α activation in a PI3K- and ERK- dependent fashion (Li et al., 2015).

Previously, we have shown that the treatment of mouse endothelial cells (MBE) with TNF results in an increase of *Adam17* mRNA levels followed by enhanced expression and activity of ADAM17 protein. Here we show that NF-κB and Elk-1 transcription factors are involved in the regulation of ADAM17 expression in MBE cells.

## RESULTS

### ADAM17 expression is increased in mouse endothelial cells exposed to IL-1β

**Fig. 1.**
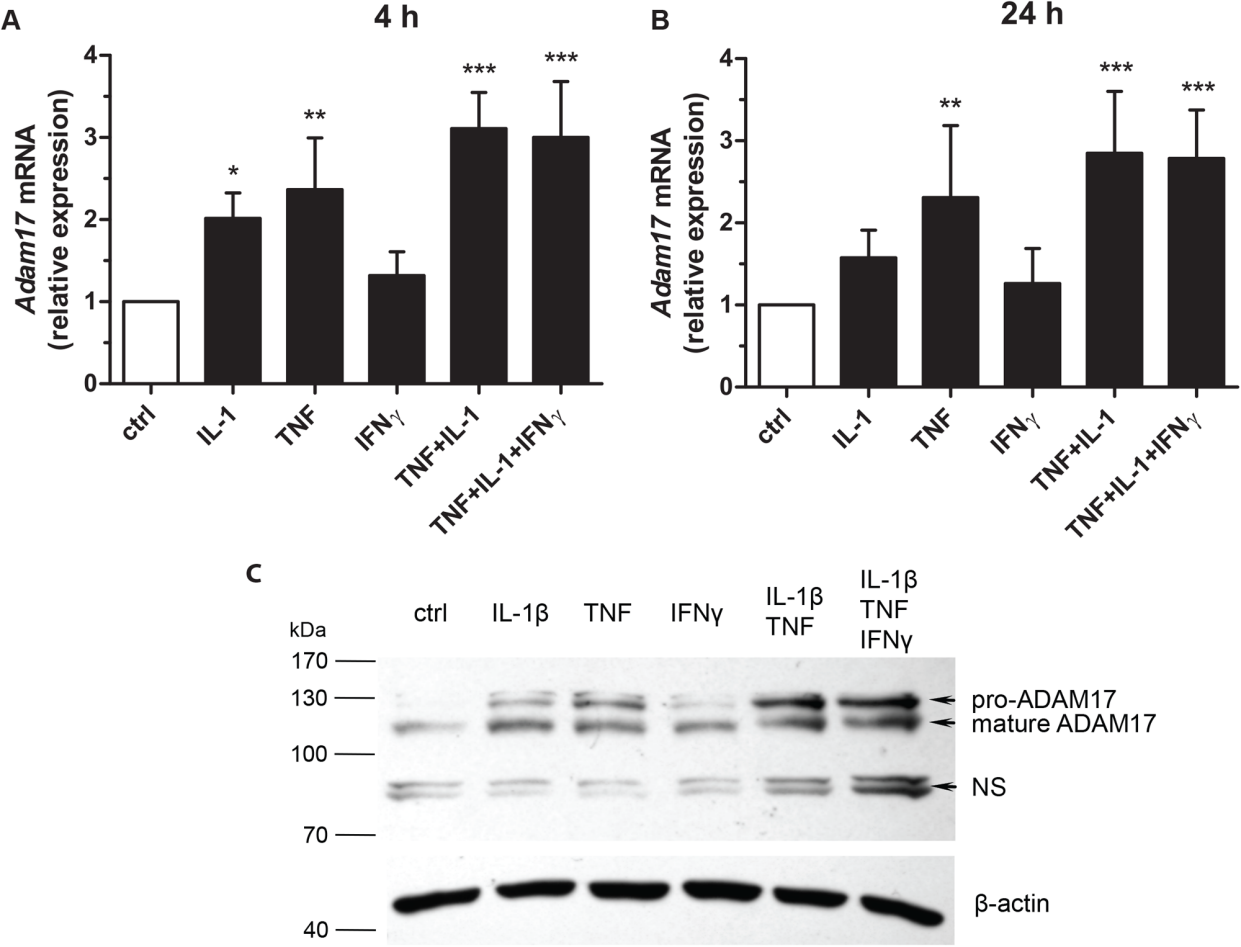
**IL-1β and TNF stimulate expression of ADAM17 in MBE cells.** MBE cells were treated for 4 h (A) or 24 h (B,C) with cytokines: IL-1β, TNF, and/or INFγ (10 ng/ml each). (A,B) *Adam17* mRNA levels were examined by qPCR. Bars represent *Adam17* mRNA levels normalized to *Eef2* mRNA levels, relative to untreated control (*n*=4, means±s.d., statistical method: one-way ANOVA with Dunnett's post hoc test, statistically significant difference versus ctrl: *P<0.05, **0.001<*P*<0.01, ***P<0.001). (C) ADAM17 protein levels were examined by western blotting. Beta-actin served as a loading control. NS, nonspecific bands. (*n*=3, a representative result).

In this study, we continued our research on the regulation of the expression of ADAM17. Using qPCR we showed that TNF or IL-1β, but not mouse IFNγ, caused an approximately twofold increase in *Adam17* mRNA levels in MBE cells after 4-h stimulation (Fig. 1A). The increased *Adam17* transcript levels in MBE in response to TNF was maintained for at least 24 h of incubation with the cytokine (Fig. 1B, 2.4-fold after 4 h, 2.3-fold increase after 24 h), confirming the results we had obtained previously using northern blotting technique (Bzowska et al., 2004). IL-1β-mediated increase in *Adam17* transcript levels was comparable to that evoked by TNF 4 h after stimulation, but it decreased faster and after 24 h was only 1.6-fold of the basal level. The simultaneous action of TNF and IL-1β resulted in a slightly higher increase in the level of mRNA for ADAM17 (3.1- and 2.8-fold, respectively, for 4 and 24 h) compared to the effect of a single cytokine. The changes in *Adam*17 mRNA levels were associated with the corresponding changes in the protein levels. After 24 h of incubation of MBE with TNF or IL-1β an increase in both the pro- and mature forms of ADAM17 was detected. It was even more pronounced when both cytokines were used simultaneously. In line with the qPCR results, no apparent effect of IFNγ on the level of ADAM17 protein in MBE cells was observed 24 h after the treatment (Fig. 1C; Fig. S1).

### The increase of ADAM17 expression is strongly affected by IKK inhibitor VII, an inhibitor of the NF-κB pathway

**Fig. 2.**
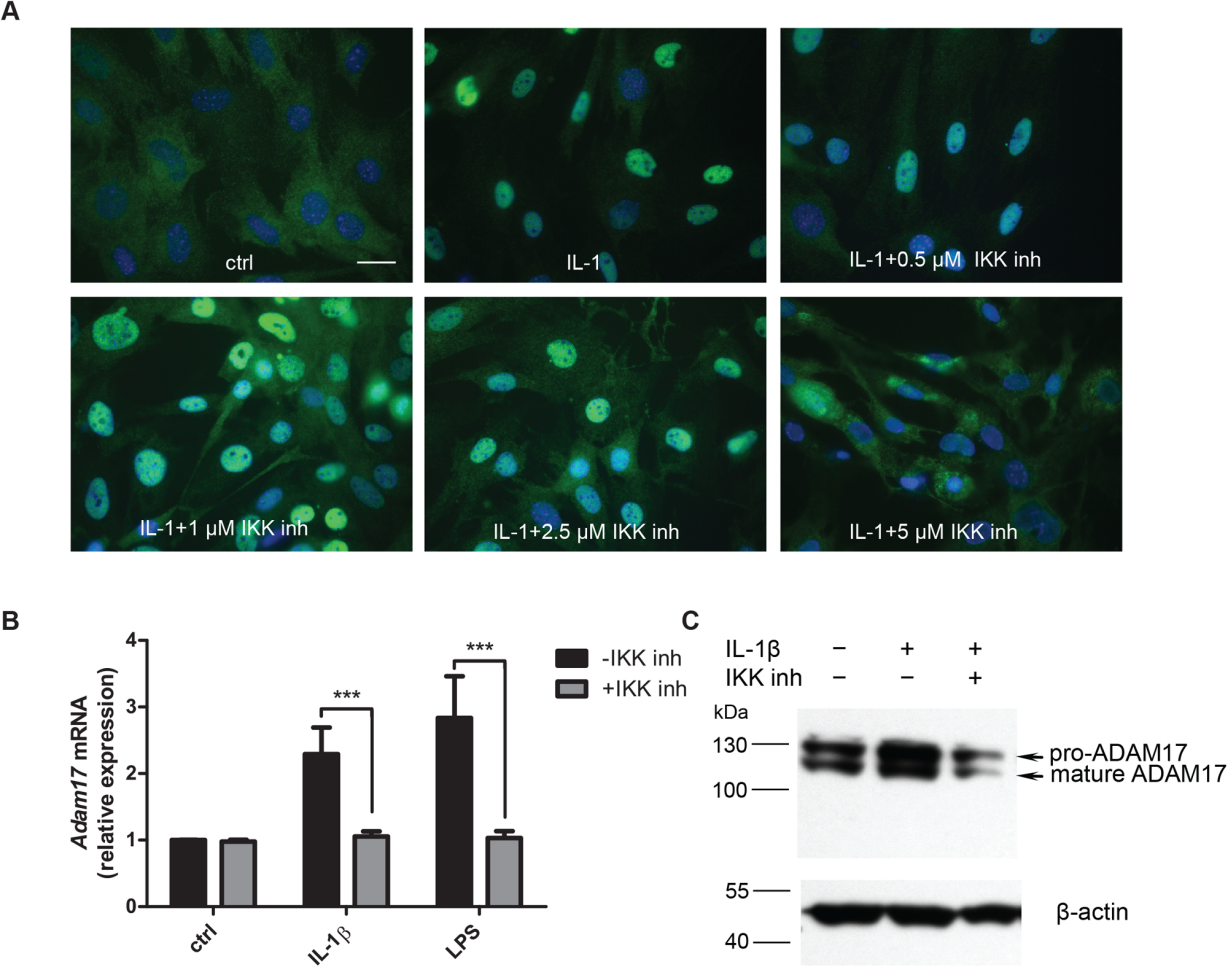
**Role of NF-κB in IL-1β- or LPS-stimulated expression of ADAM17.** (A) Analysis of IKK inhibitor VII efficiency – fluorescent microscopy. MBE cells were incubated for 1 h with IKK inhibitor VII at various concentrations (0.5 μM, 1 μM, 2.5 μM and 5 μM) and then stimulated for 0.5 h with IL-1β (10 ng/ml) or left untreated (ctrl). The cells were then fixed and stained with anti-NF-κB p65-FITC (green) and DNA-specific DAPI (blue). Scale bar: 25 μm. (B,C) Effect of IKK inhibitor VII on ADAM17 expression at mRNA (B) and protein (C) levels. After a 1 h pre-treatment with IKK inhibitor VII (5 µM) MBE cells were stimulated with IL-1β (10 ng/ml) or LPS (100 ng/ml) for 4 h (B) or 7 h (C). (B) *Adam17* mRNA levels were examined by qPCR. Bars represent *Adam17* mRNA levels normalized to *Eef2* mRNA levels, relative to untreated control (*n*=3, means±s.d., statistical method: one-way ANOVA with Dunnett's post hoc test, statistically significant difference versus ctrl: ***P<0.001. (C) Western blot analysis of ADAM17 levels. Beta-actin served as a loading control (a representative result).

Given the lack of additivity of TNF and IL-1β effects, we hypothesised that a transcription factor shared by both TNF- and IL-1β-induced signalling pathways is probably responsible for the *Adam17* transcriptional activation. One of the transcription factors that meets this criteria is NF-κB (Malinin et al., 1997). To test its involvement in regulating the *Adam17* expression we used a pharmacological inhibitor of NF-κB activation, IKK inhibitor VII, a selective inhibitor of IκB kinase. It inhibits IκB degradation and thereby prevents the cytokine-induced translocation of NF-κB from the cytoplasm to the nucleus, abrogating its activity. First, by analysing the localization of p65 subunit of NF-κB, we determined that 5 µM IKK inhibitor VII is required for the complete inhibition of NF-κB nuclear translocation in MBE. The concentration of 2.5 µM resulted in a partial inhibition (Fig. 2A). Unlike for IL-1β and LPS, the incubation of MBE with TNF in the presence of IKK inhibitor VII resulted in cell death. Therefore, we used IL-1β and LPS to examine the role of NF-κB in the stimulation of *Adam17* expression. LPS is known to activate NF-κB (Xie et al., 1994) and it also triggered the increase in *Adam17* levels in MBE (Fig. 2B). IKK inhibitor VII caused an almost complete inhibition of the IL-1β- or LPS-mediated increase in *Adam17* mRNA levels (Fig. 2B). Western blot analysis of the lysates of MBE exposed to IL-1β in the absence or presence of the inhibitor revealed that the inhibitory effect was also observed at the protein level (Fig. 2C).

**Fig. 3.**
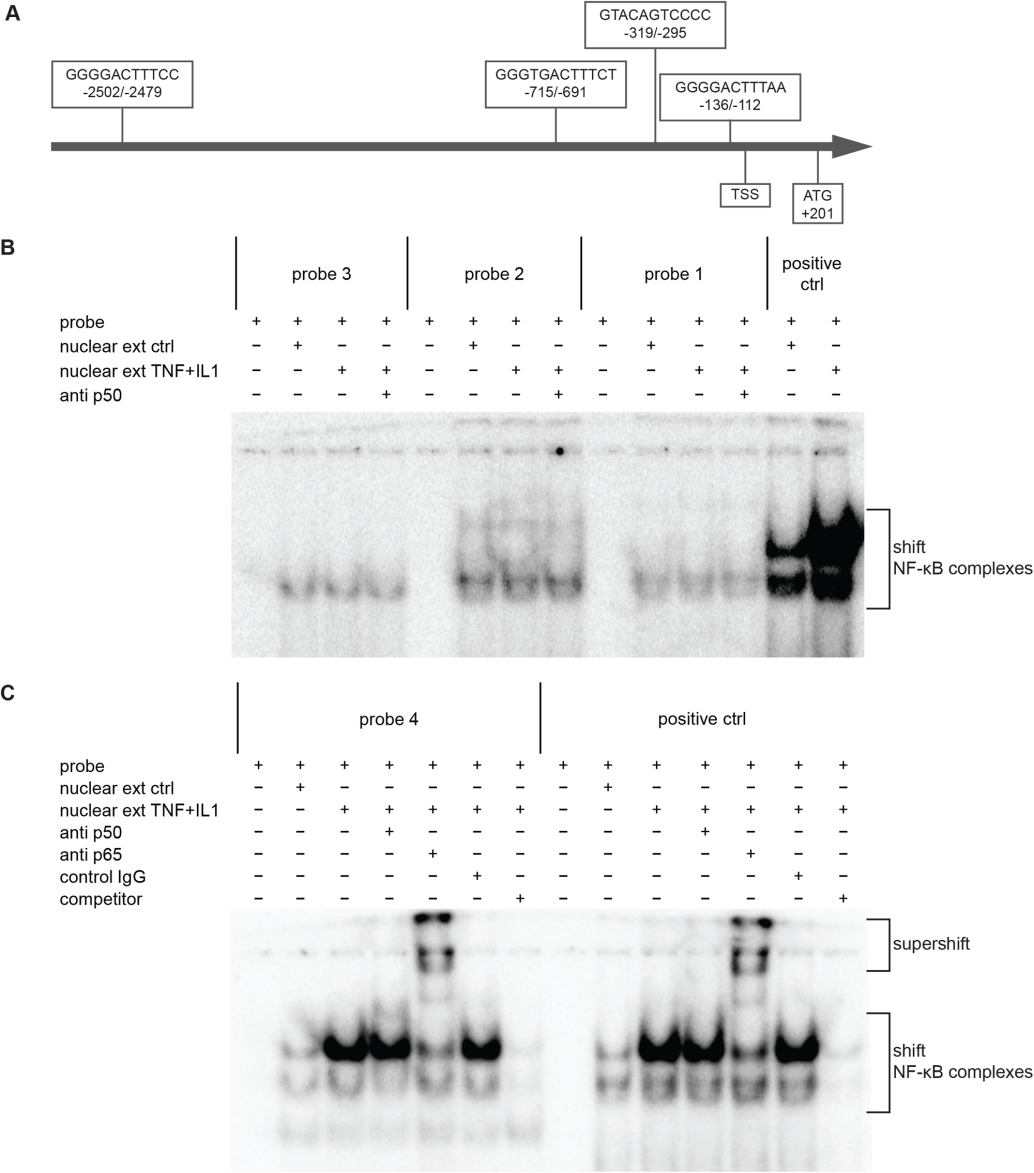
**Analysis of the interaction of selected DNA sequences within the *Adam17* promoter with NF-κB.** (A) A diagram showing the location of potential binding sites of the NF-κB in relation to transcription start site within the promoter of the mouse *Adam17* gene. (B,C) EMSA analysis of functionality of putative NF-κB binding sites in *Adam17* promoter. MBE nuclear extracts were incubated with the probes corresponding to potential NF-κB binding sequences or with a positive control probe in the absence or presence of anti-NF-κB or control antibodies. Bands in the supershift comprise complexes of p65-containing NF-κB complexes bound to the radioactive probe and anti-p65 antibody. A representative result of three independent experiments is shown.

The above experiments suggested that NF-κB plays an important role in the inflammation-stimulated *Adam17* expression. To test this hypothesis, we first searched for the potential NF-κB binding sites within the *Adam17* promoter. Using LASAGNA-Search (Lee and Huang, 2013) and Alibaba 2.1 programmes, we found four putative NF-κB binding sites within the 3.0 kb promoter region (Fig. 3A). To test their functionality, we designed indicated probes for an electromobility shift assay (EMSA; see Materials and Methods). The probe corresponding to the NF-κB site in the promoter of the mouse immunoglobulin kappa chain was used as a positive control. The results of EMSA showed that the probes 1, 2 and 3 are not capable of any substantial binding to the components of the MBE nuclear extract (Fig. 3B; Fig. S2). In contrast, probe 4 showed a strong interaction with the nuclear proteins of cytokine-stimulated MBE, and the pattern was almost identical to the one observed for the positive control (Fig. 3C; Fig. S2). The band shift caused by anti-p65 antibody indicated that the p65 subunit of NF-κB binds to probe 4. We did not identify the p65 binding partner. The antibody directed to the most likely one, p50, unexpectedly failed to delay migration of the positive control indicating that the antibody is incompatible with EMSA.

### U0126, an inhibitor of MEK1/2, reduces PMA-stimulated ADAM17 expression in MBE cells

**Fig. 4.**
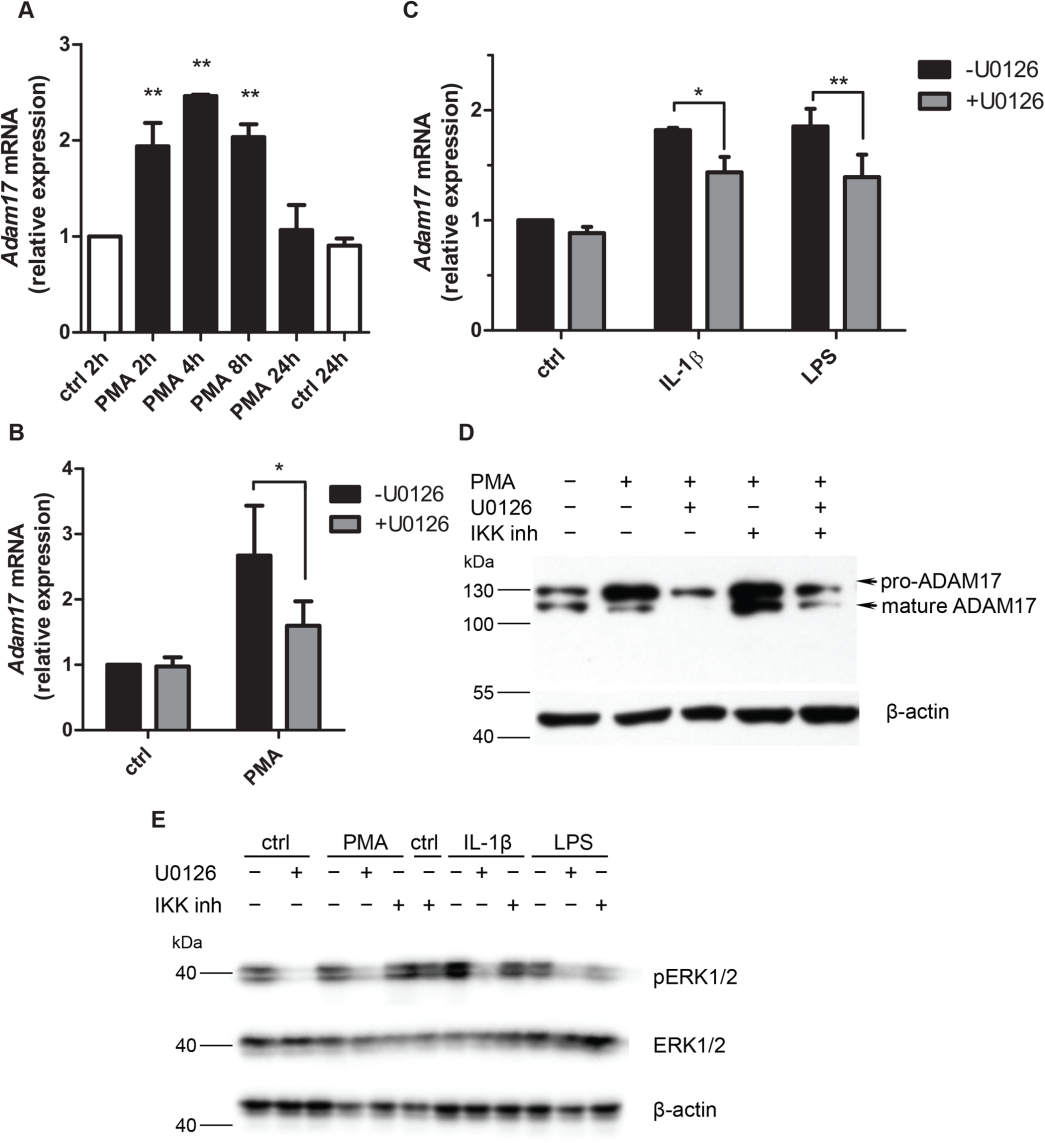
**Stimulation of ADAM17 is susceptible to inhibition by U0126, an inhibitor of MEK1/2.** (A) Kinetics of changes in *Adam17* mRNA levels in MBE treated with PMA (100 ng/ml) examined by qPCR. (B,C) Effect of U0126 (10 µM, 1 h pre-treatment) on *Adam17* mRNA levels in MBE stimulated for additional 4 h with PMA (100 ng/ml) (B) or IL-1β (10 ng/ml) or LPS (100 ng/ml), in the presence of the inhibitor (C). Bars represent *Adam17* mRNA levels normalized to *Eef2* mRNA levels, relative to untreated control [*n*=2 (A), *n*=4 (B) or *n*=3 (C)]; means±s.d.; statistical method: two-way ANOVA with Dunnett’s (A) or Bonferroni (B,C) post-test, statistically significant difference versus samples without the inhibitor: *P<0.05, **P from 0.001 to 0.01. (D) Western blot analysis of ADAM17 levels in MBE pre-treated for 1 h with U0126 (10 µM) or IKK inhibitor VII (5 µM) and for 7 h with PMA (100 ng/ml) in the presence of either inhibitor. Beta-actin served as a loading control (*n*=2, a representative result). (E) Western blot analysis of ERK1/2 phosphorylation in MBE incubated for 1 h with U0126 (10 µM) or IKK inhibitor VII (5 µM) and then for 30 min with PMA (100 ng/ml) in the presence of either inhibitor (*n*=2, a representative result).

PMA is a widely-used ADAM17 activator. We had previously demonstrated that 24 h after addition of PMA to MBE cultures there is no observable increase in *Adam17* mRNA levels (Bzowska et al., 2004). Here we showed that PMA had a short-term, stimulatory effect on ADAM17 expression. The highest *Adam17* mRNA level (2.5-fold increase versus untreated control) was observed after 4 h of PMA treatment (Fig. 4A) and was followed by a substantially elevated level of ADAM17 proform after 7 h of PMA treatment (Fig. 4D).

PMA is a well-known activator of protein kinase C (PKC) (actually of conventional and novel isoforms of the enzyme) (Robinson, 1992; Steinberg, 2008), which in turn is involved in the activation of the MAPK1/3 (ERK1/2) signalling cascade (Nakashima, 2002). To test whether stimulation of ADAM17 expression by PMA depends on ERK1/2 we used U0126, a specific inhibitor of MEK1 and MEK2, whose only known substrates are ERK1/2. The qPCR results indicated that U0126 limited the extent of PMA-mediated increase in *Adam17* mRNA levels (Fig. 4B) and ADAM17 protein levels (Fig. 4D). Unlike U0126, the NF-κB inhibitor (IKK inhibitor VII) did not influence the PMA effect on ADAM17 expression (Fig. 4D). Interestingly, U0126 caused 50% inhibition of increase in *Adam17* mRNA levels occurring in response to the physiological stimulants: IL-1β or LPS (Fig. 4C). We excluded the possibility that the observed effects of U0126 resulted from its cytotoxicity towards MBE cells because even after prolonged, 24-h incubation of the cells with the inhibitor, the metabolic activity of the cells measured by MTT test was undisturbed (data not shown). We also examined whether the 10 µM concentration of U0126 is sufficient to completely inhibit the activation of kinases ERK1/2, that is, their phosphorylation by MEK kinases. Western blot analysis showed that the U0126 inhibitor led to the inhibition of the phosphorylation of ERK1/2 after stimulation of MBE with PMA, IL-1β, or LPS (Fig. 4E). The IL-1β or LPS-induced phosphorylation of ERK1/2 was also diminished by the inhibitor of the NF-κB pathway (IKK inhibitor VII). It is not surprising as it has been shown that the activation of ERK1/2 in response to IL-1β or LPS depends on IKK (Waterfield et al., 2004). This kinase phosphorylates p105 and directs it to degradation, which results in the release of TPL-2 from the p105/TPL2 complex. TPL2 is an activator of MAP3K8, which phosphorylates MEK1/2, which in turn phosphorylates ERK1/2 (Kasza, 2013). As expected, IKK inhibitor VII did not diminish the PMA-stimulated ADAM17 levels (Fig. 4D,E).

### Elk-VP16 and Elk-En constructs regulate expression of Adam17

**Fig. 5.**
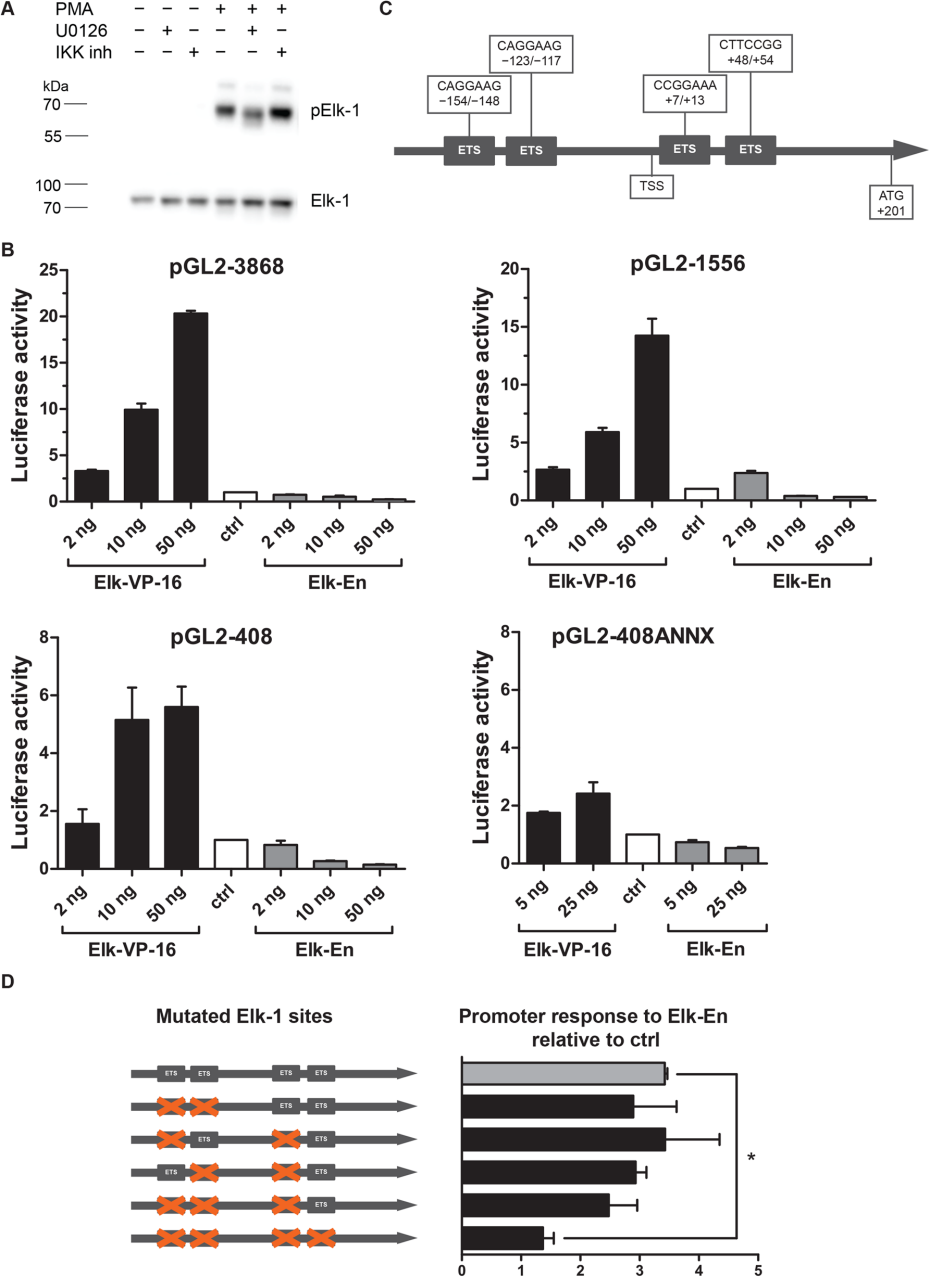
**The analysis of the interactions of Elk-1 fusion proteins, Elk-VP1 and Elk-En, with the wild-type and mutated *Adam17* promoter fragments.** (A) Western blot analysis of Elk-1 phosphorylation in MBE pre-treated for 1 h with U0126 (10 µM) or IKK inhibitor VII (5 µM) and then stimulated for 30 min with PMA (100 ng/ml) in the presence of either inhibitor. Non-phosphorylated Elk-1 served as a loading control (*n*=2, a representative result). (B) Luciferase reporter assay. MBE were transfected with one of the vectors coding for luciferase under the control of different fragments of the wild-type or mutated *Adam17* promoter concurrently with one of the plasmids encoding Elk-1 fused to an activation domain (pElk-VP16) or to a repression domain (pElk-En). Bars represent luciferase signals normalized to β-galactosidase activity, relative to the signal in control cells (*n*=3, means±s.d.). (C) A diagram showing the location of potential binding sites of Elk-1 in relation to transcription start site within the promoter of the mouse *Adam17* gene. (D) Schematic representation of 408 bp-length *Adam17* promoter constructs showing mutated potential sites of Elk-1 and their response to Elk-En (5 ng) relative to control measured in luciferase reporter assay (*n*=2, means±s.d., *P<0.05).

Our experiments with U0126 inhibitor revealed that the activation of *Adam17* transcription is in part dependent on the factors downstream of ERK1/2. Elk-1 is one of the best described transcription factors activated by ERK1/2 kinases (Wortzel and Seger, 2011). It binds *in vitro* to variants of the GGAA/T motif embedded in a larger 10-bp consensus sequence (Boros et al., 2009). Preliminary bioinformatic analysis showed that the promoter of mouse *Adam17* has several potential binding sites for this transcription factor. Using western blot analysis, we confirmed that Elk-1 became phosphorylated in MBE cells after their stimulation with PMA (Fig. 5A). Using pGL2 basic plasmid we generated three vectors, in which the firefly luciferase gene was placed under the control of the different length-fragments (3868, 1556 or 408 bp) derived from the *Adam17* promoter and we tested whether Elk–1 may indeed interact with the *Adam17* promoter. MBE were simultaneously transfected with one of the generated reporter vectors and one of the constructs encoding Elk-1 in fusion with a repressor domain (Elk-En) or with an activating domain (Elk-VP16) (Kasza et al., 2005; Price et al., 1995). Neither Elk-En nor Elk-VP16 requires phosphorylation for DNA binding (Price et al., 1995; Vickers et al., 2004). This fact was of special importance for us, as MBE cells become transiently (for at least 48 h) insensitive to stimulation after transfection (data not shown).

For all *Adam17* promoter fragments, Elk-VP16 increased and Elk-En decreased, in a dose-dependent fashion and to a similar extent, the expression of luciferase (Fig. 5B**)**. This indicates that the functional binding site(s) for Elk-1 are located within the 408 bp construct (−207 to +201 bp of the *Adam17* promoter), which is consistent with the reports indicating that the majority of potential binding sites for Elk-1 are located close to the transcription start site (Boros et al., 2009). Using LASAGNA-Search and Alibaba 2.1. tools we identified four putative Elk-1 binding sites in the *Adam17* promoter region comprising the shortest studied fragment fully responsive to Elk-VP16 and Elk-En, i.e. 408 bp (Fig. 5C).

To test the functionality of these sites, we generated a series of mutated promoter variants: four with a single mutated Elk-1 site, three with two mutated Elk-1 sites, one with three mutated Elk-1 sites and one with all four sites mutated (Fig. 5D). The analysis of luciferase activity showed that all vectors with one (data not shown), two or three mutated potential Elk-1 binding sites responded to Elk-En or Elk-VP16 in a fashion similar to the original construct lacking these mutations (Fig. 5D). In contrast, pGL2-408ANNX showed only a minimal response to the Elk-VP16 and Elk-En, suggesting that only inactivation of all four Elk-1 binding sites prevents the binding of Elk-1 to the *Adam17* promoter but also that none of these sites is essential for the Elk-1- regulated transcription of the gene controlled by this promoter.

## DISCUSSION

In this study we show increased ADAM17 expression in mouse endothelial cells (MBE) treated with TNF, IL-1β, LPS and PMA at both transcript and protein levels. We demonstrated that increased *Adam17* mRNA level can be observed as early as 4 h after stimulation. In the case of IL-1β and TNF the effect is prolonged and the increased expression lasts up to 24 h of treatment. Interestingly PMA stimulation is only temporary and cannot be observed at all after 24 h. It is possible that for sustained increased expression of ADAM17, the activation of MAP kinase pathway, occurring in response to both pro-inflammatory factors and PMA, needs to be accompanied by the activation of NF-κB pathway, observed in MBE cells exposed to IL-1β and LPS but not to PMA. PMA is known to be a potent inducer of ADAM17-mediated shedding activity. However, following ADAM17 activation, the cells treated with PMA display decreased ADAM17 expression on the cell surface; this effect can be observed as early as 30 min after stimulation (Doedens et al., 2003) and results from PMA-mediated ADAM17 internalization and degradation. The form of ADAM17 that is preferentially downregulated in response to PMA, is the mature form of the protein (Doedens and Black, 2000; Lorenzen et al., 2016). From the homeostatic point of view it is beneficial that PMA, which stimulates ADAM17 shedding activation and internalization, at the same time stimulates ADAM17 expression as a feedback mechanism, which allows to restore the mature form of ADAM17 at the cell surface. However, the link between ADAM17 internalization and its potentiated expression is not yet recognized.

Takamune et al. showed that TNF is able to induce maturation of ADAM17. They observed that the TNF-mediated conversion of the ADAM17 proform to its mature form was blocked by an inhibitor of NF-κB transcription factor in oral squamous carcinoma cells (Takamune et al., 2008). TNF was also shown to increase *ADAM17* mRNA level in a NF-κB-dependent manner. The authors suggest that TNF, being an ADAM17 substrate, upregulates itself through inducing ADAM17 expression and maturation (Takamune et al., 2008). We confirmed that NF-κB is necessary for LPS- and IL-1β-induced ADAM17 expression, because the increase of *Adam17* mRNA stimulated by these factors was completely blocked by an NF-κB inhibitor. Additionally, we identified the sequence in *Adam17* promoter, which is responsible for the binding of p65 subunit of NF-κB. The EMSA probe corresponding to the sequence containing NF-κB consensus site located in *Adam17* promoter at position −2502 to −2479 relative to the transcription start site (TSS) was able to bind to p65 present in nuclear extracts of cytokine-stimulated MBE cells. Such a significant distance of the identified functional NF-κB from TSS may explain the moderate increase of *Adam17* expression in response to cytokines and LPS. It has been shown that NF-κB strongly stimulates expression of its target genes only if its binding site lies within 200 bp upstream of TSS; if NF-κB binding site is located further upstream, the magnitude of stimulation is in the range of 1.6–2.6, which is in perfect agreement with our results (Tabach et al., 2007). We also verified that three additional putative NF-κB-binding sequences in *Adam17* promoter are not functional.

Using MEK inhibitor U0126 Takaguri et al. showed that ERK is a critical molecule in IL-1-induced ADAM17 expression in vascular smooth muscle cells (Takaguri et al., 2016). In our study involving endothelial cells, the substantial, yet incomplete inhibition of PMA- as well as IL-1β- and LPS-induced ADAM17 expression by U0126 suggests that the extent of ERK involvement in the regulation of ADAM17 expression may be cell- or species-specific. Transcription factor Elk-1 is a well-known nuclear ERK1/2 target (Wortzel and Seger, 2011). Elk-1-binding regions are located mainly around TSS, and most of them within 1 kb of the TSS (Boros et al., 2009). Using several deletion constructs of the *Adam17* promoter, we showed that Elk-1 binding occurs within −207 to +201 bp fragment of the promoter. The site-directed mutagenesis of putative Elk-1 binding sites allowed us to demonstrate the role of Elk-1 in the regulation of *Adam17* transcription. Interestingly, the exact site of Elk-1 binding within this region seems not to be essential because Elk-1 effect on activation of *Adam17* promoter was prevented only by mutating of all four Elk-1 binding sites.

Taken together, this report shows that both NF-κB and Elk-1 transcription factors play a crucial role in the regulation of ADAM17 expression in mouse endothelial cells.

## MATERIALS AND METHODS

### Cell culture and transfection

MBE were a gift from R. Auerbach (University of Wisconsin, USA). MBE were cultured in DMEM high glucose (Lonza, Switzerland) supplemented with 10% foetal bovine serum (FBS) (Biowest, France) in a humidified atmosphere at 37°C with 5% CO_2_. The cells were routinely checked for mycoplasma contamination by PCR as described previously (Kochan et al., 2016). MBE were seeded in 24-well plates (5×10^4^ cells in 0.5 ml medium per well) and transfected with indicated vectors using Lipofectamine 2000 (Invitrogen) according to the manufacturer's instruction. Where indicated, the cells were stimulated with 10 ng/ml rhIL-1β (PromoKine, Germany), 10 ng/ml rhTNF (PromoKine), 10 ng/ml rmIFNγ (PromoKine), 100 ng/ml PMA (Sigma-Aldrich) or 100 ng/ml LPS (Sigma-Aldrich). Where required, the inhibitors: 5 µM IKK Inhibitor VII (Merck, Germany) or 10 µM U0126 (Cell Signaling Technology) were added 60 min prior to stimulation.

### Plasmid construction

*Adam17* promoter regions (comprising 408, 1556, 3868 bp upstream of the translation start site, i.e. located at −207 to +201, −1355 to +201, and −3667 to +201 in relation to TSS, respectively), were amplified by PCR using genomic DNA isolated from MBE cells, cloned into pTZ57R vector (Fermentas, Lithuania) and sequenced to confirm their identity to *Adam17* promoter sequence deposited in NCBI (accession number NC_000078.6). They were next cloned into pGL2-basic luciferase reporter vector (Promega) resulting in pGL2-408, pGL2-1556 and pGL2-3868 plasmids. Mutations of potential Elk-1 binding sites (ETS Mut) were introduced into the sequence of murine *Adam17* promoter using site-directed mutagenesis. A modified QuikChange-based mutagenesis technique was used to introduce point mutations within the potential binding sites for Elk-1. QuikChange^®^ Primer Design programme was used to design the primers. PCR conditions: 95°C, 2 min; 17 cycles: 95°C, 40 s; 60°C, 1 min; 72°C, 2 min/kbp; and final extension, 72°C for 2 min. pGL2-408 was used as a template and amplified with *Pfu* DNA polymerase (Fermentas, Lithuania). The product was digested with *Dpn*I and then used for bacterial transformation. Positive clones were selected based on the presence of the cleavage site for the appropriate restriction enzyme (*Aat*II, *Nco*I, *Nru*I or *Xho*I) introduced with the mutagenic primers: MElkAat–GCCGCCAGCTGAAGGCCGACGTCGCGCCAAGCAGGCCCCA, MElkAatR–TGGGGCCTGCTTGGCGCGACGTCGGCCTTCAGCTGGCGGC MElkNru–AGCAGGCCCCAGAGGGACCTCGCGAGAACAGAGCCCAGAAGATG, MElkNruR–CATCTTCTGGGCTCTGTTCTCGCGAGGTCCCTCTGGGGCCTGCT, MElkNco–GGTCTCCGGCTGCGGCCATGGACGAGTTAAGCCGCTCT, MElkNcoR–AGAGCGGCTTAACTCGTCCATGGCCGCAGCCGGAGACC, MElkXho–GCGAGCGCCGCCTGCACTCGAGGGGACGTGA, MElkXhoR–TCACGTCCCCTCGAGTGCAGGCGGCGCTCGC, and then sequenced. Repeated rounds of mutagenesis were used for preparation of the constructs, in which several potential binding sites for Elk-1 have been mutated.

### Total RNA extraction, reverse transcription and qPCR

The total RNA was isolated using the Chomczynski method (Chomczynski and Sacchi, 1987). 1 μg of total RNA was reverse transcribed using oligo(dT)_15_ and M-MLV Reverse Transcriptase (Promega). qPCR was performed using KAPA SYBR FAST qPCR Kit (Kapa Biosystems, USA). The primers for *Adam17* (mAdam17RT_F–CCAGGAGCGCAGCAACAAGGT, mAdam17RT_R–TCCTATCACTGCACTGCACACCCG) and for the reference gene *Eef2* (EF2L–GCGGTCAGCACACTGGCATA, EF2R–GACATCACCAAGGGTGTGCAG) were from Genomed, Poland. Primer efficiencies (95–100% range) were assessed using the serial dilution method. The thermal cycling conditions included: an initial denaturation step at 95°C for 10 min, and then 40 cycles: 95°C for 10 s, 60°C for 15 s and 72°C for 20 s. The experiments were carried out in duplicates for each data point. The relative quantification of gene expression was determined using the ΔΔCt method (Livak and Schmittgen, 2001). RNA integrity was determined with agarose gel electrophoresis under denaturing conditions.

### Western blotting

MBE were seeded in a six-well plate at a density of 2×10^5^ cells per well. Next day the medium was replaced with fresh DMEM supplemented with 0.5% of FBS. 24 h later the cells were treated with stimulants (IL-1β, TNF, LPS, PMA). The inhibitors, IKK inhibitor VII and U0126, were added 1 h before stimulation. The cells were lysed with RIPA buffer (50 mM Tris-HCl pH 8.0, 150 mM NaCl, 1% NP-40, 0.5% DOC, 0.5% SDS) containing cOmplete™ protease inhibitor cocktail (Roche, Switzerland). Protein concentrations in lysates were measured with the BCA method using the BCA Protein Assay Kit (Bicinchoninic Acid Kit, Sigma-Aldrich). Western blot analysis was performed according to a standard protocol (Mahmood and Yang, 2012), with primary antibodies: rabbit anti-ADAM17 (ab2051, Abcam, diluted 1:5000), rabbit anti-β actin (13E5, Cell Signaling Technology, diluted 1:5000), rabbit anti-ERK1/2 (#9102, Cell Signaling Technology, diluted 1:1000), rabbit anti-pERK1/2 (#9101, Cell Signaling Technology, diluted 1:1000) and peroxidase-conjugated goat anti-rabbit IgG secondary antibody (A6667, Sigma-Aldrich, diluted 1:40,000). Membranes were imaged with the Fusion-Fx documentation system (Vilber Lourmat). The adjustments of the images of membranes, which involved cropping and whole image contrast scaling, were performed using Quantity One (Bio-Rad).

### Electromobility shift assay (EMSA)

MBE (1.6×10^6^ cells) were seeded in 100-mm culture plates in 10 ml medium. The following day the medium was replaced with fresh DMEM supplemented with 0.5% of FBS. 48 h after seeding, the cells were stimulated with IL-1β and TNF. After 30 min the cells were rinsed with ice-cold PBS and collected with a rubber scraper. The cell pellets were incubated on ice in a buffer containing 10 mM Hepes pH 7.8, 10 mM KCl, 0.1 mM EDTA, 0.1 mM EGTA, 1 mM Na_3_VO_4_, 1 mM dithiothreitol, 0.2 mM phenylmethanesulfonyl fluoride and cOmplete™ protease inhibitor cocktail (Roche, Switzerland). After 10 min 0.7% ND-40 was added and the samples were centrifuged for 3 min at 13,000× ***g***, 4°C. The nuclear extracts were obtained by the incubation of the nuclei for 15 min on ice in a buffer containing 20 mM Hepes pH 7.8, 400 mM NaCl, 1 mM EDTA, 1 mM EGTA, 1 mM Na_3_VO_4_, 1 mM dithiothreitol and cOmplete™ and removal of debris via centrifugation (5 min, 14,000× ***g***, 4°C). Protein concentrations were measured using the BCA method. The nuclear extracts were stored at −80°C as a 10% glycerol solution. Four potential NF-κB binding sites in the promoter region of *Adam17* were found using LASAGNA-Search (Lee and Huang, 2013) and Alibaba 2.1 (http://gene-regulation.com/pub/programs/alibaba2/) programs available online and the probes covering the identified sequences were designed (Table 1). Double stranded DNA probes were obtained by mixing equimolar quantities (5 μM each) of appropriate oligonucleotide pairs, heating them to 90°C for 5 min and leaving them to renature by slowly cooling down at room temperature (RT). The renaturated dsDNA probes (1 pmol) were radiolabelled with 10 μCi of ³²P by incorporating [α-³²P]dCTP (Hartmann Analytic, Germany) during a 3′ fill-in reaction using Klenow exo^−^ fragment (Fermentas, Lithuania). The labelled probes were purified with QIAquick Nucleotide Removal Kit (Qiagen). The samples of nuclear extracts (5 µg) were incubated for 5 min with 1 µg of poly [d(I–C)] in the binding buffer (10 mM Hepes, pH 7.8, 100 mM NaCl, 0.5 mM EDTA, 10% v/v glycerol and 0.2 mM dithiothreitol). Additionally, some of the samples contained 0.4 µg of anti-p65 (Santa Cruz Biotechnology), anti-p50 (sc-114, Santa Cruz Biotechnology) or control rabbit IgG (10500C, Thermo Fisher Scientific). Next, 1 µl of the purified radiolabelled probe was added to each sample and incubated for 30 min at RT. 5% non-denaturing TBE-polyacrylamide gel was used to resolve protein-DNA complexes from free DNA. Electrophoresis was run at 160 V for 1.5 h in 0.5× TBE. The gel, after vacuum drying, was placed on a phosphoimager screen and read after 24 h using Molecular Imager and Quantity One (Bio-Rad).

**Table 1.**
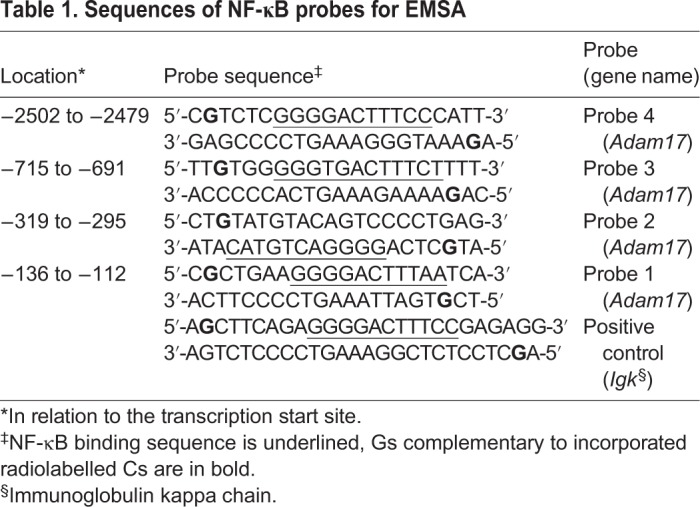
**Sequences of NF-κB probes for EMSA**

### Immunofluorescence

MBE were seeded on glass coverslips (15×15 mm, Knittel Glasbearbeitungs, Germany) in six-well plates at a density of 2.0×10^5^ cells/well in 2.5 ml complete medium. The following day the medium was changed to 0.5% FBS and 24 h later cells were treated with varying concentrations of IKK inhibitor VII (Merck, Germany) and/or IL-1β or left untreated. Subsequently the cells were washed with PBS and fixed with 4% formaldehyde in PBS for 15 min at RT. Next the cells were washed two times with PBS, blocked/permeabilised for 60 min at RT in blocking buffer (5% FBS, 0.3% Triton X-100 in PBS) and incubated overnight at 4°C in a humidified chamber with anti-p65 antibodies (SC-372X, Santa Cruz Biotechnology) diluted 1:1000 in blocking buffer. The following day, the cells were washed with PBS and incubated in the dark for 60 min at RT with DyLight550-conjugated anti-rabbit antibody (ab98489, Abcam) diluted 1:150 in blocking buffer. Following incubation the cells were washed three times with PBS and the coverslips were mounted onto slides in VECTASHIELD Mounting Medium with DAPI (Vector Laboratories, USA). The samples were imaged using a Leica DM IRE2 fluorescence microscope equipped with a digital black and white CCD camera. The images were converted to RGB colours in the ImageJ 1.48v program (NIH).

### Luciferase reporter assay

Plasmids pElk-VP16 and pElk-En were a gift from Prof. A. Sharocks (University of Manchester, UK). MBE cells were co-transfected with (i) varying amounts of pElk-VP16 or pElk-En (2, 10 or 50 ng), and (ii) 200 ng of pGL2-408 or pGL2-1556 or pGL2-3868, and (iii) 5 ng of pEF (internal vector control encoding β-galactosidase) and pcDNA3 in such an amount as to equalize to 800 ng of total DNA per well. Transfections were done in duplicates in 24-well plates. In 24 h after transfection the cells were lysed, firefly luciferase and β-galactosidase activities were measured using Dual-Light Luciferase and β-Galactosidase Reporter Gene Assay (Thermo Fisher Scientific). The light emissions were measured with a 20/20^n^ luminometer (Turner Biosystems, USA). For each sample the luciferase activity was normalized to the reporter β-galactosidase activity.

### Statistical analysis and graphs

All graphs and statistical analyses were done using GraphPad Prism (version 5.0, GraphPad Software, USA). The exact tests applied are indicated in figure descriptions.

## Supplementary Material

Supplementary information
