# Supplementary material for: The role of NF-κB and Elk-1 in the regulation of mouse ADAM17 expression
Source: Biol Open. 2019 Feb 15;8(2):bio039420. doi: 10.1242/bio.039420 (PMC6398470; doi:10.1242/bio.039420)
Supplement: Supplementary information [file biolopen-8-039420-s1.pdf]

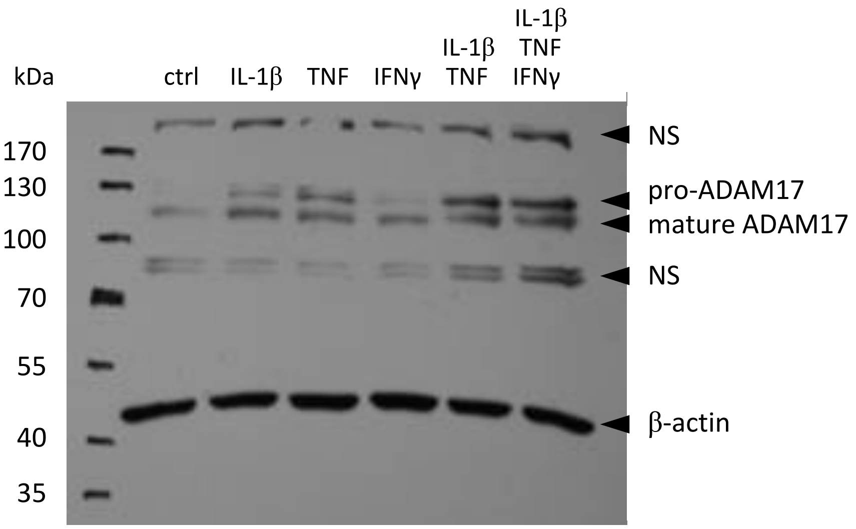

Fig. S1. Original image of Western blotting presented on Fig. 1C in the main text. After transfer the membrane was cut and ADAM17 and β-actin were probed separately. Next the fragments of the membrane were put together and jointly subjected to chemiluminescence reaction and analysis.

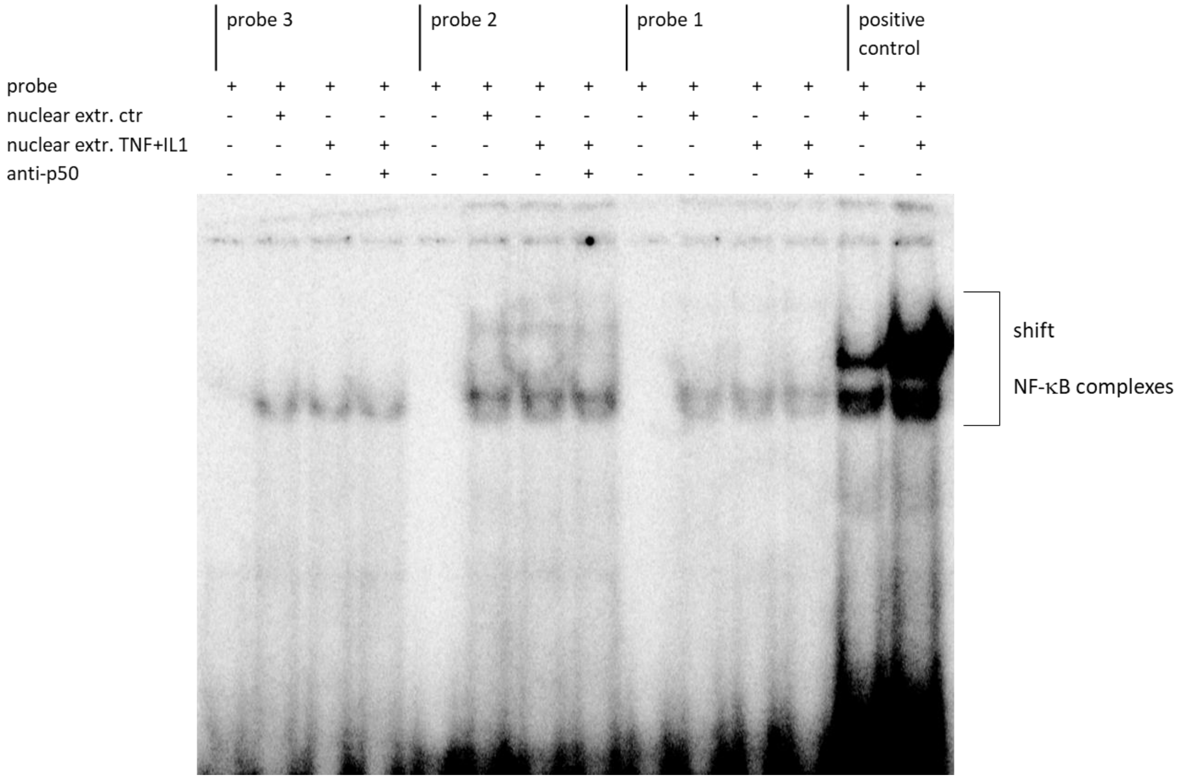

Biology Open • Supplementary information

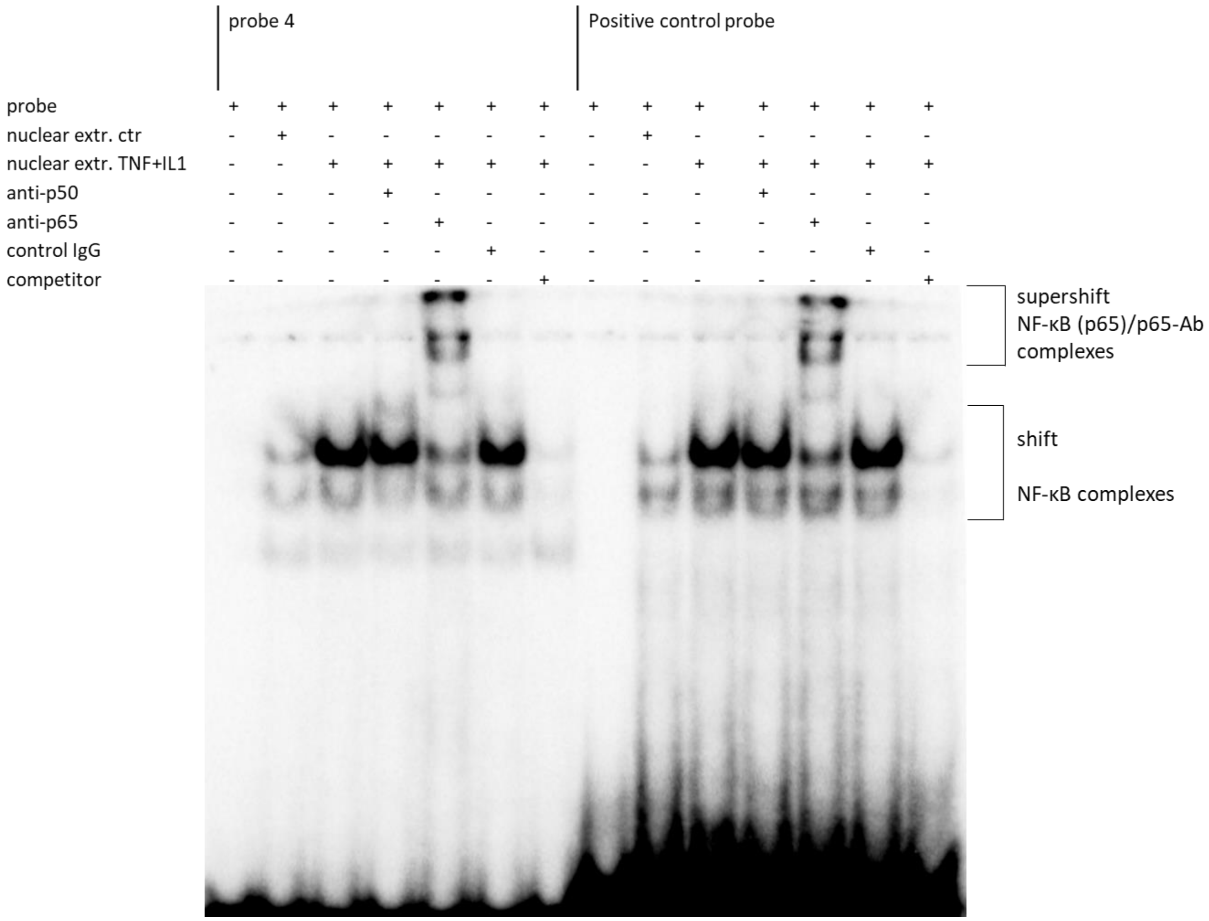

Fig. S2. Original images of EMSA analysis presented on Fig. 3B and C in the main text.
